# Exploring Effects of Household Air Pollution on Pregnant Mothers and Their Offspring in Africa: A Scoping Review

**DOI:** 10.3390/ijerph23030363

**Published:** 2026-03-12

**Authors:** Livhuwani Muthelo, Mxolisi Welcome Ngwenya, Joyce Shirinde, Tebogo Maria Mothiba

**Affiliations:** 1 Department of Nursing Science, University of Limpopo, Polokwane 0727, South Africa; 2 School of Health Systems and Public Health, University of Pretoria, Pretoria 0028, South Africa; 3 Faculty of Health Science, University of Limpopo, Polokwane 0727, South Africa

**Keywords:** implications, pregnant, women, and household air pollution

## Abstract

**Highlights:**

**Public health relevance: How does this work relate to a public health issue?**

- This scoping review addresses household air pollution as a critical public health concern that leads to preventable mortality and morbidity in pregnant women, resulting in negative pregnancy outcomes and long-term health implications for both mothers and their children.

**Public health significance: Why is this work of significance to public health?**

- Household air pollution is a potentially modifiable environmental risk factor that leads to adverse health outcomes for mothers and their children. The findings from this review are important for guiding public health efforts to protect pregnant women and lower unnecessary illness and death rate.

**Public health implications: What are the key implications or messages for practitioners, policymakers, and/or researchers in public health?**

- The findings highlight the need to integrate household air pollution screening, risk awareness, and clean household energy interventions into maternal and prenatal health programmes, with policies that prioritize low-income households and gender-sensitive approaches.

**Abstract:**

In recent decades, air pollution has been the cause of major mortality and morbidity worldwide. WHO attributes about 4.2 million of these to ambient pollution and 3.2 million to household sources. Pregnant women are no exception to those mortalities. Therefore, this review aims to explore and critique existing evidence on the implications of household air pollution in homes among pregnant women. In this review, we adhered to the 2018 PRISMA Scoping Review guidelines. We followed the iterative steps. The time horizon of the literature was 2014–2024. The literature search was conducted on databases such as ProQuest, ScienceDirect, EBSCOhost, and PubMed. Only 19 publications met the inclusion criteria and were critically analyzed using thematic analysis technique. The review yielded two themes: (1) practices that predispose pregnant women to household air pollution and (2) impacts of household air pollution on the health of pregnant women. The study highlighted that socioeconomic status and gender roles play a vital role in exposure to air pollution among pregnant women. Therefore, this review finds it vital for future research to directly examine the impact of socioeconomic factors on air pollution. There is a particular need to develop strategies to mitigate air pollution in the African context. Furthermore, this review recommends that future research also focuses on the long-term biological effects of air pollution among pregnant women.

## 1. Introduction

In recent decades, air pollution has emerged as the leading cause of major morbidity and mortality worldwide. Air pollution is a major global health threat, contributing to an estimated seven million deaths each year [1]. WHO attributes about 4.2 million of these to ambient pollution and 3.2 million to household sources. Its 2021 Air Quality Guidelines set health-based limits that highlight the preventable burden of air pollution [1]. Despite efforts by the United Nations to ensure health, well-being and a safe environment for all, air pollution does not seem to receive adequate attention. Nearly 3 billion people across the globe rely on the use of polluting fuels for cooking and heating [2]. Countries such as Brazil and India have to move towards cleaner energy to mitigate air pollution, through using liquified petroleum gas. This is mainly because it mitigates air pollution emissions [3].

African countries still struggle with measuring and maintaining air quality. Approximately 1.1 million deaths were reported in Africa, including 697,000 caused by household air pollution (HAP) in 2019 [4]. It seems that pregnant women are no exception to this risk. It has been identified by the study of Esong et al. [5] that women are more at risk of exposure to HAP than other populations. This may be due to their gender-specific roles, such as spending most of their time indoors cooking and taking care of their families. This was indicated by a study in Ethiopia that about 75% of women use solid fuels, such as firewood, for cooking [5,6]. These household practices include burning solid waste that emits air pollutants such as carbon monoxide, particulate matter (PM_2.5_ and PM_10_), sulfur dioxide and nitrogen oxide [7]. These air pollutants impact the health and well-being of pregnant women. In many African countries, limited waste management infrastructure and inadequate transportation systems contribute to practices such as the open burning of household waste [8]. Rather than reflecting individual awareness, this practice largely results from structural limitations in waste collection services. Improving municipal waste systems and expanding collection capacity could reduce open burning and improve air quality. Several studies highlighted the health impacts of those practices on the population and pregnant women involved [9,10]. Among those health impacts are respiratory problems, low birth weight, and stillbirths [9,10]. While there is considerable evidence regarding the health impacts, little is known about the biological mechanisms by which air pollution affects pregnancy and fetal development, particularly in low and middle-income countries. Thus, in this review, we plan to delve into detail on how air pollution affects socioeconomic factors and the biological mechanisms of pregnancy [10].

Drawing from the literature, it seems that a substantial gap remains on how socioeconomic status intermingles with air pollution, consequently affecting pregnancy [11]. Therefore, it is worth noting that most African countries, such as countries like Ghana, Congo, Zimbabwe, and Sudan, are classified as low- and middle-income countries, where poverty persists [11]. Above all, some African countries have high unemployment rates and fertility rates of greater than 4.02 births per woman [12]. Amidst all that, there are educational disparities between women and men in Africa, with most men holding higher levels of education than women [13]. The struggles of unemployment and educational disparities among women often result in them remaining at home to care for their families, thereby increasing their risk of exposure to higher levels of HAP. Despite growing evidence on HAP, there remains limited understanding of how socioeconomic factors specifically shape pregnant women’s exposure in African settings. Adopting the scoping review methodology, the authors were able to obtain a broad understanding of the topic under study, using a diversity of the relevant literature and studies of different methodologies [14]. Therefore, this scoping review aims to synthesize the existing literature to clarify these socioeconomic determinants and identify gaps that require further research. For these reasons, this explores and critiques the existing evidence on the implications of HAP among pregnant women.

## 2. Methodology

This study followed the Arksey and O’Malley scoping review methodology. Tricco et al. [14] substantiated that the use of scoping reviews allows researchers to identify knowledge gaps and set research agendas and implications for decision-making. The scooping review was not registered with Prospero or any platform.

### 2.1. Research Questions

The subsequent research question steered this review.

- What evidence is available on the effects of household air pollution on pregnancy outcomes in Africa?

### 2.2. Relevant Literature Search

The literature search was initiated from July 2024 to August 2024. The first and second authors conducted a search of literature from the various databases. The databases used were PubMed, EBSCOhost, ScienceDirect, and Proquest. The search strategy adopted by the authors was the use of keywords and Boolean operators such as “AND” to find all the literature on HAP in the home and pregnant women in Africa. Quotation marks and asterisks were used to narrow the literature search to the phenomenon under study. However, to expand the literature search, the authors further conducted backward and forward referencing and grey literature searching to gather all the existing evidence on HAP and pregnant women. The literature was identified using the subsequent keywords in the databases.

Keywords: Household air pollution, indoor air pollution, pregnant women, maternal health, Africa, health outcomes, prenatal exposure.

Search string examples: (“household air pollution” OR “indoor air pollution”)

AND (pregna* OR antenatal OR prenatal OR “pregnant women” OR “maternal health”)

AND (Africa OR “African countries”)

AND (implication* OR impact* OR effect*)

PubMed: (pregnant women) OR (prenatal) OR (antenatal) OR (maternal health) OR (gravid women) AND (y_10[Filter]) AND (exclude preprints [Filter]) AND (English [Filter]) AND (Indoor air pollution) OR (household air pollution) OR (indoor air quality) AND (y_10 [Filter]) AND (exclude preprints [Filter]) AND(English [Filter]) AND (Africa) OR (African countries) OR (Namibia) OR (Nigeria) OR (Ethopia) OR (Egypt) OR (DR Congo) OR (Tanzania) OR (South Africa) OR (Kenya) OR (Sudan) OR (Uganda) OR (Algeria) OR (Angola) OR (Morocco) OR (Mozambique) OR (Ghana) OR (Madagascar) OR (Cote d’Ivoire) OR (Cameroon) OR (Niger) OR (Mali) OR (Burkina Faso) OR (Malawi) OR (Zambia) OR (Chad) OR (Somalia) OR (Senegal) OR (Zimbabwe) OR (Guinea) OR (Benin) OR (Rwanda) OR (Burundi) OR (Tunisia) OR (South Sudan) OR (Togo) OR (Sierra Leone) OR (Libya) OR (Congo) OR (Liberia) OR (Central African republic) OR (Gabon) OR (Lesotho) OR (Botswana) OR (Eswatini) OR (Swaziland) OR (Equatorial Guinea) OR (Mauritania) OR (Cabo Verde) OR (Seychelles) OR (Comoros) AND (y_10 [Filter]) AND (exclude preprints [Filter]) AND (English [Filter])

### 2.3. Study Selection and Eligibility

**Figure 1:**
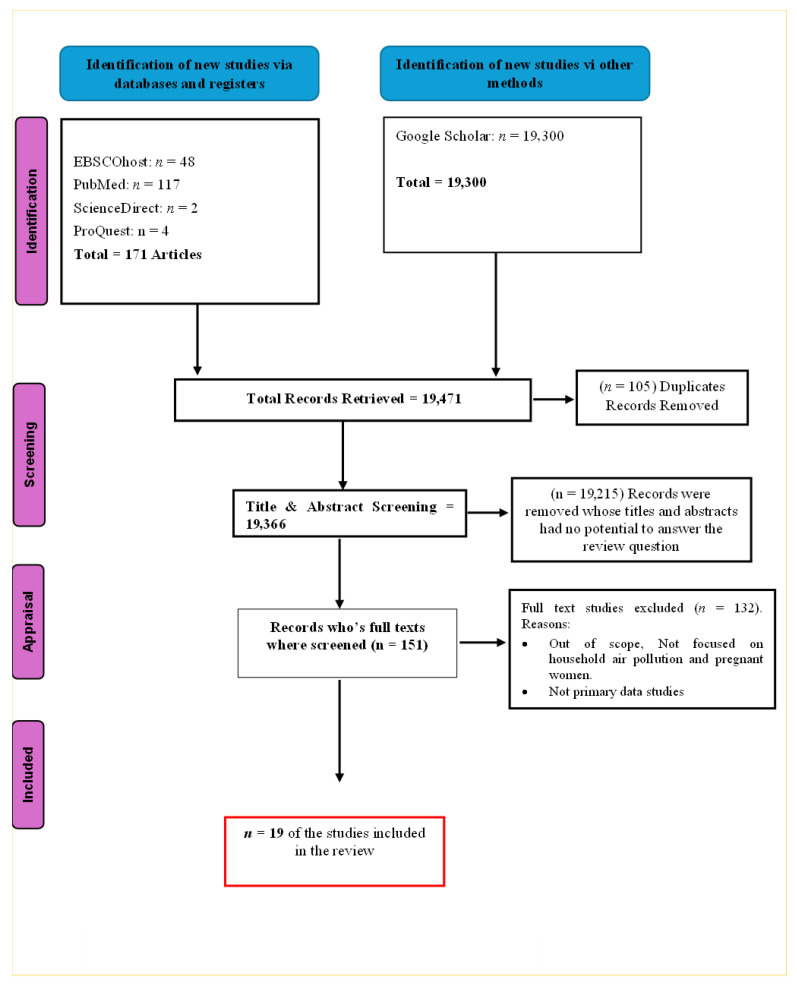
Flowchart of the study selection.

The literature search looked at publications between January 2014 and June 2024. The search from the mentioned databases yielded 19,471 publications. The authors then initiated the literature screening. The screening began with the title and abstract screening to determine if the publications were relevant to the phenomenon of inquiry. Throughout the screening, the authors kept in mind the inclusion criteria of the review. The publications that met the inclusion criteria were subjected to a full-text review by all the authors independently to identify the similarities and prominent findings across the publications. English-written qualitative, quantitative, and mixed-method studies and clinical trials were included in this review. Publications on air pollution and COVID-19 in pregnant women were excluded from this review. Publications not addressing the phenomenon of inquiry, literature reviews, and editorials were excluded from this review. Following the identification and screening processes, only 19 publications met the inclusion criteria. Publications that looked at the HAP exposure were included. Table 1 and Figure 1 show the eligibility criteria of the review and study selection process, respectively.

### 2.4. Charting the Data

The authors designed a data extraction tool in Microsoft Excel^TM^. The data extraction tool captured the authors, publication date, study design, country, sample size, type of exposure, and assessment characteristics and outcomes. The designed tool was refined by all the reviewers, who reached a consensus on the items to be captured by the tool, following piloting of the tool using about five studies. The authors assessed the tool to determine whether it extracts all the variables required to answer the study question. Thereafter, the authors (LM and MW) independently proceeded to data extraction from the 19 publications that met the inclusion criteria. Table 2 depicts the characteristics of the selected studies.

### 2.5. Collate, Summarize, and Report the Results

Considering that the data extracted was captured in Microsoft Excel^TM^, the authors resorted to the use of descriptive statistics to represent the characteristics of the eligible publications. However, we analyze the studies in-depth. Thematic analysis was used to analyze the data from the selected publications. The analysis was guided by the six iterative steps of the thematic analysis framework by Braun and Clarke [33]. The authors read through the eligible publications multiple times to familiarize themselves with the publications. This assisted the authors in identifying similarities and differences between the publications. The publications with similar conceptual meanings and views were coded and clustered together to identify any potential themes. The authors arranged consecutive meetings following the independent analysis to reach agreements and consensus on the potential themes. Any disagreements and discrepancies among the potential themes were resolved through discussion.

## 3. Results

The literature search yielded 19,471 publications. Following the screening and removal of duplicates, only 151 publications were thoroughly reviewed, and 19 publications were selected. The full text and thorough review of the publications yielded two themes.

### 3.1. Characteristics of the Included Publications

The publications that were included in this contemporary review range from 2015 to 2024, most of the publications were quantitative studies (84.2%), followed by qualitative studies (10.5%), and mixed-method studies are the least prevalent at 5.3%. Most of the studies came from Ghana (36.8%), followed by studies from Nigeria (21.1%) and South Africa (21.1%), and then those from Tanzania (10.5%) and Ethiopia (10.5%). This is displayed in Table 3 below.

### 3.2. Practices That Predispose Pregnant Women to Household Air Pollution

**Figure 2:**
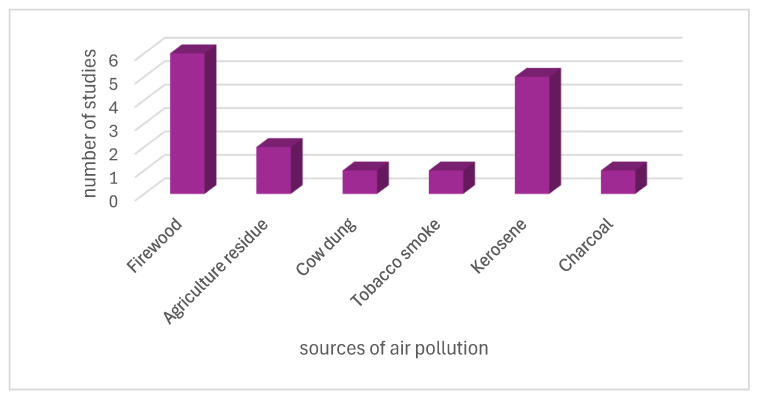
Sources of air pollution.

Figure 2 presents the primary sources of HAP for pregnant women, including firewood, agricultural residue, cow dung, tobacco smoke, kerosene, and charcoal, all of which have been shown in studies to emit pollutants that can increase health risks during pregnancy.

The use of biomass is a prominent theme among pregnant women across the studies reviewed here. Figure 2 above indicates that 32% of the 19 studies reported that pregnant women use firewood for household chores such as cooking, whilst 26% of the studies indicated that the pregnant women used Kerosene for cooking [15,16,18,21,23,24,25,26,28]. Cow dung and charcoal were the least used at 5% [16,21]. A recent quantitative analysis by Weber et al. [21] in Ghana reported that approximately 34% of 819 pregnant women use polluting solid fuels for cooking, such as wood, charcoal, and crop residue. Comparably, a study by Van Vliet et al. [24] reported that 94% of pregnant women from rural communities predominantly use wood for cooking, followed by charcoal at 4%. These practices were similar across the countries, such as Ethiopia, South Africa [16,18]. This may have been due to chance or might reflect a socioeconomic effect. Among the socioeconomic attributes, sociodemographic factors such as level of and employment status influence the use of polluting fuels for cooking among pregnant women [16,18,21]. The study of Weber et al. [21] further concedes that most pregnant women with low educational level and no formal employment were more likely to use polluting fuels. Also, the exposure to HAP seems to be attributed to gender-specific roles. In many African rural communities, traditional gender roles often place the responsibility of cooking and household fuel use on women, which can increase their exposure to indoor air pollution. Looking at the socioeconomic status of rural communities, most of the pregnant women in rural Ghana and those enrolled to the DCHS, in a low-socioeconomic, peri-urban South African community, resorted to the use of electrical power-saving cooking approaches such as burning firewood for cooking [18,24].

However, the study of Habtamu et al. [16] pointed out that some pregnant women are aware of air pollution exposure impacts. This was indicated by pregnant women who perceived air pollution to be associated with respiratory problems and major health outcomes such as asthma, sneezing, coughing, abortion, and low birth weight [28]. The study of Shezi et al. [15] concedes that although pregnant women are aware of the impact of HAP, there are still some women who adopt practices predisposing pregnant women enrolled to the DCHS, in a low-socioeconomic, peri-urban South African community, to air pollution exposure during pregnancy. Among these are incense burning and indoor smoking. The reasons reported for these practices continuing included spousal negligence, financial instability, and poor knowledge level among pregnant women [28].

However, some studies noted that tobacco smoking exposure is exposure to HAP. It is a common problem in rural areas. The study identified some pregnant women smoke actively and others are passive smokers. This may be due to living with smoking husbands and relatives [15]. This was also evident in a study by Van Vliet et al. [24] that indicated that approximately 21% of pregnant women are exposed to passive smoking in their households. The review ideally suggests that cultural and gender norms increase the exposure to HAP among pregnant women, prejudicing them to poor pregnancy outcomes.

### 3.3. Implications of Exposure to Household Air Pollution Among Pregnant Women

The literature review revealed prominent health issues that occur during exposure to household air pollution among pregnant women. These health issues arise from exposure to particular matter, volatile organic compounds, and carbon monoxide. These health issues were classified as implications for maternal health, fetoplacental unit, infant development, and pregnancy outcomes. This is discussed in detail below.

#### 3.3.1. Implications of Household Air Pollution Exposure for Maternal Health and Fetoplacental Unit

The findings of this review suggest that exposure to particulate matter and other air pollutants pose significant risks to maternal and fetal health. This was evident in a clinical trial conducted by Alexander et al. looking at clean-energy and non-clean-energy use by pregnant women, which indicated that exposure to biomasses that produce air pollution, such as Kerosene and firewood is significantly associated with blood pressure changes over time, particularly diastolic blood pressure (*p* = 0.040) [20]. They further indicated that about 6.4% of pregnant women who used kerosene had hypertension more than those who used clean energy such as ethanol stoves [23]. Despite the limitations of the sample size, it is worth considering that exposure to HAP has an immense negative impact on maternal health.

However, exposure to HAP also impacts the respiratory health of pregnant women. In Ghana, 94% of pregnant women used firewood for cooking, and the reported prevalent symptoms among the pregnant were phlegm with a cough at 9.6%, followed by coughing (6.2%), wheezing (4.8%) and dyspnea (4.6%) [24]. The study indicated a positive association between wheezing, dyspnea, and coughing for more than five days, as well as exposure to air pollution like carbon monoxide.

This literature indicates that exposure to HAP negatively impacts fetal development. It was noted by the study of Kaali et al. [25] that prenatal exposure to HAP has negative implications for fetal well-being. The study indicated that increased exposure to HAP alters the cord blood mononuclear cell mitochondrial DNA copy number and reduces it [25], consequently leading to prenatal oxidative stress injury [25]. One could infer that once a prenatal oxidative stress injury occurs, the fetoplacental unit will be consequently affected. Kaali et al. [25] suggests that this consequently causes decreased head circumference and gestational age at birth. On the other hand, Dutta et al. [26] bemoans that exposure to HAP and clean stove energies has no significant differences in fetal growth biometers such as head and abdominal circumference and femur length. Despite the six repeated ultrasonographies at various prenatal care visits by pregnant women, no significant changes were observed.

Meanwhile, two studies conducted in Nigeria and Tanzania deplored that although there are no significant differences, HAP affects the placenta and adversely impacts fetal well-being [19,26]. The study conducted in Nigeria indicated that HAP alters chronic placental biomarkers [27]. This was substantiated by the significant increase in placenta biomarkers (Hofbauer cells (HBC), syncytial knots (SK), and chorionic vascular density (cVD)) with the use of firewood and kerosene for cooking by pregnant women, and hypoxia-inducible factor (HIF) was consistently associated with chronic hypoxia among those women [27]. The study conducted in Tanzania further corroborated that exposure to HAP exposure results in placental pathology and is significantly associated with fetal thrombotic vasculopathy among pregnant women exposed to household pollutants such as particular matter and carbon monoxide [19]. It seems that exposure to air pollution might impact fetal well-being as hypoxia means the fetus will not be receiving enough oxygen and this may result in adverse pregnancy outcomes [19].

#### 3.3.2. Implications of Household Air Pollution Exposure on Pregnancy Outcomes and Infant Development

A study conducted in South Africa has revealed that exposure to HAP has implications for pregnancy outcomes [15]. The study concedes that although HAP has negative implications during fetal development, it is also associated with low birth weight and preterm labour with the odds ratio of low birth weight and preterm delivery being 1.75 and 1.21, respectively, per interquartile increase (18 µg/m^3^) in PM_2.5_ exposure. [15]. This was concurred by a study conducted in Tanzania that also indicated that exposure to HAP results in adverse pregnancy outcomes such as low birth weight [19]. A study in Nigeria also conceded that HAP has adverse effects on pregnancy outcomes. Although no statistical association was found between the measured variables, the study indicated high rates of preterm deliveries, miscarriages, stillbirths, and perinatal mortalities among pregnant women using kerosene and firewood stoves [20]. The existing evidence suggests that the adverse pregnancy outcomes such as low birth weight are not only limited to one geographical context in Africa. This was also reported in Tanzania, Nigeria and South Africa [15,19,20]. Among pregnancy outcomes, Weber et al. [21] reported low Apgar score at 5 min and perinatal mortality as the consequent outcomes of exposure to air pollution. Thus, the clinical trial of Alexander et al. recommended the use of clean-energy stoves, such as ethanol-stove use, because it will reduce poor pregnancy outcomes among pregnant women.

However, from the literature, it seems that prenatal exposure to HAP does not only impact the fetus’s health in utero. Prenatal exposure to HAP further impacts the child’s health in the early years of life. Several authors indicated that after delivery, the infant exposed to HAP in utero had impaired lung function during the first few years of life [22,29]. On the ordeal of impaired lung function, Kinney et al. [30] indicated that exposure increases the risk of pneumonia by 10% (relative risk [RR], 1.10; 95% CI, 1.04–1.16) and severe pneumonia by 15% (RR, 1.15; 95% CI, 1.03–1.28), per 1 part per million (ppm) increase in average prenatal CO exposure and by 6% (RR, 1.06; 95% CI, 0.99–1.13) per 1 ppm increase in average postnatal CO exposure, during the first year of life among infants who were prenatally exposed to HAP. Drawing from the literature, it is worth considering that the health implications that air pollution has on exposed children are beyond the respiratory system only. It was also indicated in several studies throughout Africa that exposure to HAP results in impaired neurological development, poor infant growth, and higher blood pressure levels among children. Christensen et al. indicated that exposure to HAP pollutants impairs neurological development particularly concerning the cognition, language and adaptive behaviour among children under two years [17,31,32].

### 3.4. Exposure to Pollutants and Health Implications

**Figure 3:**
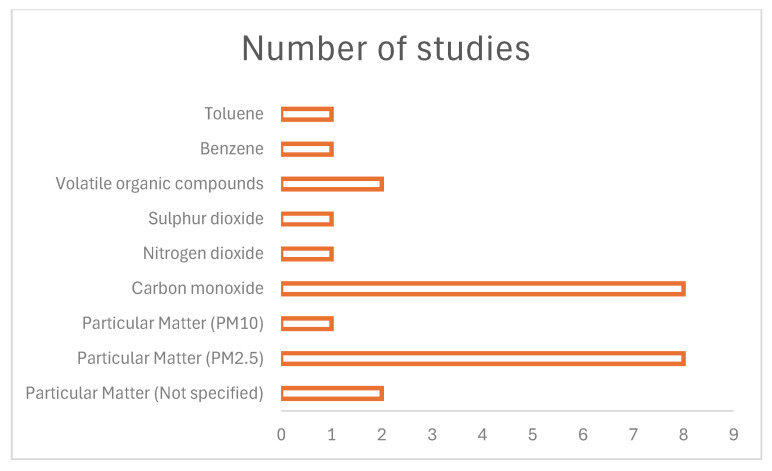
Type of exposure.

Figure 3 shows that PM_2.5_ and carbon monoxide are common measured pollutants in Ghana, South Africa, Tanzania, and Nigeria [15,18,22,24,25,26]. The pollutants are linked to the burning of solid biomasses such as kerosene, firewood, and agricultural residuals. The studies identified that carbon monoxide and PM_2.5_ increases blood pressure and the risk of respiratory diseases among pregnant women. For instance, a study in Ghana indicated that exposure to carbon monoxide and PM_2.5_ increases a risk of pneumonia and severe pneumonia [30]. In support of this, our review also indicated that these pollutants impair lung function [29].

In the same wavelength, Figure 3 shows that toluene, benzene, sulphur dioxide, PM_10_ and nitrogen dioxide were the least measured pollutants [29]. These findings suggest there is a need to measure the level of household exposure to these pollutants among pregnant women. This will assist towards identifying the health implications that these pollutants have on maternal and fetal well-being.

## 4. Discussion

This scoping review explores the available literature on the health effects of household air pollution among pregnant women and their offsprings in Africa. Four major themes emerged, namely, practices that dispose pregnant women to household air pollution, implications of exposure to household air pollution among pregnant women and exposure to pollutants and health implications. Our findings identified that the majority of the pregnant women were using firewood and kerosene for cooking purposes. These practices were similar across other countries and were not only limited to pregnant women. For instance, a study in Bangladesh reported that about 90% of pregnant women use solid biomasses for cooking purposes [34]. The practices of using solid biomass waste were observed mostly in low- and middle-income countries. Also, a study conducted in Bangladesh, Pakistan, Chile, Colombia, India and China reported that most of the populations used firewood for cooking purposes [35]. In Lesotho, about 39.6% of people used firewood as the primary cooking source [36]. The choice of using these solid biomasses was influenced by socioeconomic status. This review briefly highlighted how socioeconomic and demographic disparities influence behaviour and living conditions that contribute to air pollution exposure. This was evident from several studies that indicated that education level, age, affordability and culture impact the choice of cooking fuel, thus impacting air pollution practices [5,16,21]. Limited knowledge of the health risks of household air pollution may influence women’s use of biomass fuels, indoor cooking practices, and inadequate ventilation, thereby increasing exposure to smoke and harmful pollutants. Other authors admit that socioeconomic factors, such as level of education, negatively influence women’s ability to find and understand due to limited access to information, which shapes their decision-making about the type of cooking fuel [37,38]. This dependence increases exposure to harmful pollutants associated with household air pollution. A study conducted in Uganda further reported that poverty and low educational attainment were associated with increased risk of diseases linked to household air pollution [39]. While educational interventions may improve awareness, their impact is likely limited if cleaner fuels such as LPG and electricity remain unaffordable, highlighting the need for government support through subsidies and improved access to clean energy. Although, some studies suggest differences in household air pollution between rural and urban areas, Esong et al. [5] reported no significant difference, likely due to high levels of poverty and informal settlements in both environments. This was also confirmed by the pregnant women’s perspectives that highlighted that a lack of knowledge among pregnant women could be one of the reasons women continue with practices that predispose them to household air pollution exposure [28]. However, several studies looked at how the vulnerable population of pregnant women, and their practices of air pollution, are putting them at risk. Several studies highlighted that the prominent practice of exposing women to high air pollution exposure, particularly in rural communities, is the burning of biomass for cooking, heating, and burning of incense [5,28].

In interpreting these household practices, it is important to acknowledge the physiological differences between gravid and non-gravid women. During pregnancy, the whole-body system is bound to change. These include increases in cardiovascular function, changes in respiratory systems such as increased oxygen demand, and immunological changes in pregnant women that place them at increased risk and severity of certain diseases [40,41]. Along with this physiological adaptation and air pollution exposure practices, pregnant women are at risk. Other studies have said that women are the housekeepers of the households, this is understood as the social role of women. They assume duties such as cooking and tending to their families. They assume prominent practices such as the burning of biomasses for cooking, heating and the burning of incense, particularly in rural areas [16]. This was supported by several studies that involved a larger sample size of women, indicating that most of the women relied on the robust use of firewood, kerosene, sawdust, and charcoal for household chores [5,15,16]. These prominent practices were indicated to be influenced by various factors, among which are affordability, availability, rapidity, culture, and ease of use [5]. An interesting finding of the review is that socioeconomic factors influence the practices of pregnant women. However, there is a substantial gap in the relationship between sociocultural and gender norms with exposure to household air pollution in the African context. This review briefly highlighted sociodemographic factors that impact exposure to air pollution, such as the level of education and employment status [5,16]. Little is known about the impact of sociocultural and gender norms on exposure to air pollution. Despite the circumstances, this review advocates for in-depth scientific evidence that looks at the roles of pregnant women and their impact on air pollution. Additionally, scientific longitudinal studies look at the effects of exposure to air pollution during the entire pregnancy, from the early first trimester to the last trimester. These findings can assist researchers in developing strategies to address and transform the cultural norms that predispose vulnerable pregnant women to air pollution exposure, thereby reducing the adverse effects of pollution on both the fetus and the mother. This instigates the global saying ‘it takes a village to raise a child.’ By this, the authors urge the involvement of societies and communities to partake in the desensitization of practices promoting air pollution that puts the feto-maternal well-being in danger, thus achieving the Sustainable Development Goals (SDG) SDG 3.1 (reducing global maternal mortality), SDG 3.2 (ending preventable deaths of newborns and children under five years of age), and SDG 3.9 (reducing deaths and illnesses attributable to air, water, and soil pollution and contamination) [42]. It is pivotal for future studies to consider sociocultural norms in Africa to understand the prolonged exposure to air pollution among pregnant women.

Our findings also indicated that exposure to pollutants causes adverse effects on maternal well-being and pregnancy outcomes. Several of the studies indicated health conditions such as coughing, wheezing, increased risk of pneumonia, and increased blood pressure [6,9,24,30,32]. The other implications of air pollution involve low birth weight and perinatal mortality [10,20]. Our findings are consistent with the existing literature, a study in Bangladesh affirms that the household air pollution increases the risks of neonatal mortality and low birth weight [34]. Our findings linked the health implications to the exposure of PM_2.5._ and carbon monoxide from the use of solid fuels. A multinational study conducted in low- and middle-income countries revealed that the use of firewood increases PM_2.5_ tenfold [35]. The constant exposure to the PM_2.5_ is further associated with increased risk of gestational hypertension, respiratory illness, preterm labour, and low birth weight [43]. Other studies have shown one in seven pregnant women exposed to household air pollution have at least one adverse pregnancy outcome. These were specifically due to exposure to particular matter, kerosene, and biomass fuel [44].

Another interesting finding from the review is that exposure to household air pollution pollutants also affects the fetal development and infant growth. Our review highlighted that users of firewood and kerosene elevates the placental biomarkers. Specifically, Hoffbauer cells responsible for maintaining a healthy pregnancy [45]. The exposure to PM_2.5_ increased the Hoffbauer cells, SK and cVD, leading to oxidative stress causing chronic hypoxia to the placenta [27]. These findings are similar to a study conducted in the Netherlands by Hooven et al. [46] that indicated that exposure to air pollution pollutants is affects the biomarkers of placental growth and function. These findings indicate a relationship between household air pollution exposure and placental growth. Therefore, we suggest that further studies be conducted to investigate in-depth the impact of such exposure on the feto-placental unit.

Our scoping review has limitations which pertain to the scope of the available literature and inclusion criteria. The reviewed studies had limitations which pertained to methodology, which focused on the short-term effects of household air pollution rather than long-term implications. Future research should aim to address these gaps by conducting longitudinal studies with larger populations to better understand the chronic impacts of household air pollution on maternal and fetal health. It is against this limitation that this review contends with the necessity of longitudinal scientific research among pregnant women to mitigate the health implications as a result of air pollution. Due to the explorative and descriptive nature of the scooping review, the study focused on mapping the existing evidence on implications of air pollution in pregnancy, rather than explaining the cause-and-effect relationship between the exposure levels and health implications. Therefore, future research should be conducted to explain the causal and effect relationship between the exposure levels and health implications. Some of the included studies relied on self-reported data of the symptoms and health implications of the HAP which may subject the study to social desirability bias and underreporting of the health implications of air pollution.

## 5. Conclusions

The review revealed that household air pollution remains a public health challenge with critical health implications on pregnant women and fetus well-being. Exposure to household air pollutants harms fetal development resulting in low birth weight, neonatal mortality, and an increase in the risk of developing hypertension, pneumonia in the mother, and impaired lung function. The review further highlighted that socioeconomic factors are the crucial elements that predispose pregnant women to air pollution. As a result, pregnant women used solid fuels for cooking, not because of preference but out of necessity. Our findings illuminate that poverty and socioeconomic status shapes the household energy choices. Therefore, this review finds it vital for future research to directly focus on the impact of socioeconomic factors on air pollution to develop sustainable household energy strategies to mitigate air pollution in the African context. Furthermore, the review recommends that future research should also focus on the long-term biological effects of air pollution among pregnant women.

## Figures and Tables

**Table 1:** Inclusion criteria for studies.

Inclusion criteria	Primary and peer-reviewed studies (qualitative, quantitative, and clinical trials).	Exclusion criteria	Secondary studies such as reviews and editorials (narrative, scooping, and systematic).
	Studies on household air pollution and its health impacts.		Studies not focused on household air pollution
	Studies involving pregnant women.		Studies involving populations other than pregnant women
	Studies focused on African countries.		Studies focused outside the African continent
	Studies published in English.		Studies not published in English.

**Table 2:** Characteristics of the selected studies.

Author and Publication Year	Title of Study	Research Method	Target Population and Sample Size	Country	Type of Exposure/Sources of Pollutants	Main Study Findings
Shezi et al. (2021) [15]	Maternal exposure to indoor PM_2.5_ and associated adverse birth outcomes in low socioeconomic households, Durban, South Africa	Quantitative method	Pregnant women (N = 800)	South Africa	Particular matter (PM_2.5_)	Low birth weight (LBW, defined as birth weight < 2500 g) and preterm delivery.
Habtamu et al. (2023) [16]	Health risk perceptions of household air pollution and perceived benefits of improved stoves among pregnant women in rural Ethiopia: a mixed method study	Mixed method	Pregnant women (N = 455)	Ethiopia	Firewood Agriculture residuals Cow dung	The study indicated awareness of pregnant women of household air pollution. And covered factors that influence the adoption of household air pollution practices.
Christensen et al. (2022) [17]	In-utero exposure to indoor air pollution or tobacco smoke and cognitive development in a South African birth cohort study	Quantitative method	Mothers and babies (N = 734)	South Africa	Tobacco smoke Particular matter (PM_10_)	Prenatal exposure to PM_10_ and tobacco associated with neurological development among children at two years, particularly cognition, language and adaptive behaviour.
Vanker et al. (2015) [18]	Home Environment and Indoor Air Pollution Exposure in an African Birth Cohort Study	Quantitative method	Mothers and babies (N = 633)	South Africa	Particular matter Sulphur dioxide Nitrogen dioxide Carbon monoxide Volatile organic compounds	Higher levels of indoor air pollutant were also associated with winter compared to autumn, spring and summer. Low socioeconomic status and poor home environments were prevalent and contributed to higher indoor air pollution levels. The study reported significant association between cooking with fossil fuels with increased benzene, carbon monoxide and nitrogen dioxide.
Wylie et al. (2017) [19]	Maternal exposure to carbon monoxide and fine particulate matter during pregnancy in an urban Tanzanian cohort	Quantitative method	Pregnant women (N = 239)	Tanzania	Carbon monoxide Particular matter	Low birth weight.
Alexander et al. (2018) [20]	Pregnancy outcomes and ethanol cook stove intervention: A randomized controlled	Quantitative method	pregnant women (N = 324)	Nigeria	Kerosene Firewood	The study concedes that HAP has adverse effects on pregnancy outcomes. Although no statistical association was found between the measured variables, the study revealed high rates of preterm deliveries, miscarriages, stillbirths and perinatal mortalities among pregnant women using kerosene and firewood stove.
Weber et al. (2020) [21]	Household fuel use and adverse pregnancy outcomes in a Ghanaian cohort study	Quantitative method	Pregnant women (N = 1010)	Ghana	Firewood Charcoal Kerosene Crop residue	Solid fuel burning was statistically associated with perinatal mortality and low Apgar scores at 5 min.
Lee et al. (2019) [22]	Prenatal Household Air Pollution Is Associated with Impaired Infant Lung Function with Sex-Specific Effects. Evidence from GRAPHS, a Cluster Randomized Cookstove Intervention Trial	Quantitative method	Mothers and infants (N = 384)	Ghana	Carbon monoxide	Impaired lung function for 30-days-old infant was reported in this study. It was further highlighted that there is increased risk of pneumonia in the first year of life.
Alexander et al. (2017) [23]	Randomized controlled ethanol cookstove intervention and blood pressure in pregnant Nigerian women	Quantitative method	pregnant women (N = 324)	Nigeria	Kerosene Firewood Ethanol stoves	The change in diastolic blood pressure (DBP) over time was significantly different between ethanol users and control subjects (using kerosene and firewood) (*p* = 0.040). 6.4% of control subjects were hypertensive (SBP ≥ 140 and/or DBP ≥ 90 mm Hg) versus 1.9% of ethanol users (*p* = 0.051).
Van Vliet et al. (2019) [24]	Current respiratory symptoms and risk factors in pregnant women cooking with biomass fuels in rural Ghana	Quantitative method	pregnant women (N= 840)	Ghana	Carbon monoxide	Prominent respiratory symptoms among the pregnant women were wheezing, coughing, and dyspnoea.
Kaali et al. (2018) [25]	Prenatal Household Air Pollution Alters Cord Blood Mononuclear Cell Mitochondrial DNA Copy Number: Sex-Specific Associations	Quantitative method	mother-infant (N = 120)	Ghana	Carbon monoxide	Using a polymerase chain reaction to measure mitochondrial deoxyribonucleic acid copy number from cord blood. This study identified that exposure to carbon monoxide reduces the cord blood mitochondrial deoxyribonucleic acid copy number causing prenatal oxidative injury.
Dutta et al. (2021) [26]	Household air pollution, ultrasound measurement, foetal biometric parameters, and intrauterine growth restriction	Quantitative method	pregnant women (N = 324)	Nigeria	Firewood Kerosene Particular matter (PM_2.5_)	This is no significant association between PM_2.5_ and fetal parameters such femur length, abdominal circumference, head circumference, biparietal diameter, and ultrasound estimated foetal weight.
Wylie et al. (2016) [19]	Placental Pathology Associated with Household Air Pollution in a Cohort of Pregnant Women from Dar es Salaam, Tanzania	Quantitative method	239 placentas (Pregnant women)	Tanzania	Particular matter (PM_2.5_) Carbon monoxide	Fetal thrombosis may contribute to the adverse outcomes associated with household air pollution from cook stoves during pregnancy.
Dutta et al. (2018) [27]	Household air pollution and chronic hypoxia in the placenta of pregnant Nigerian women: A randomized controlled ethanol Cookstove intervention	Quantitative method	36 placenta samples	Nigeria	Firewood Kerosene Particular matter (PM_2.5_) Ethanol stoves	Hofbauer cells (HBC), syncytial knots (SK), and chorionic vascular density (cVD) were significantly increased among firewood/kerosene users compared to ethanol users and natural gas users in Chicago. Hypoxia-inducible factor (HIF) expression consistent with chronic hypoxia in placenta of firewood/kerosene users compared to ethanol users with less HAP exposure and Chicago women with no presumed HAP exposure.
Shine et al. (2023) [28]	Pregnant women’s perception on the health effects of household air pollution in Rural Butajira, Ethiopia: a phenomenological qualitative study	Qualitative method	15 pregnant women	Ethiopia	Not Applicable	The pregnant women in this study had associated HAP exposure with respiratory problems such as coughing, sneezing and asthma. Pregnant women also mentioned HAP exposures causes major problems like asphyxia, abortion, low birth weight, and hydrocephalus.
Chaya et al. (2023) [29]	The impact of antenatal and postnatal indoor air pollution or tobacco smoke exposure on lung function at 3 years in an African birth cohort	Quantitative method	Infants (N= 798)	South Africa	PM_2.5_ Volatile organic compounds Benzene Toluene	Impaired lung function.
Kinney et al. (2021) [30]	Prenatal and Postnatal Household Air Pollution Exposures and Pneumonia Risk: Evidence from the Ghana Randomized Air Pollution and Health Study	Quantitative method	pregnant women and infants (N = 1141)	Ghana	PM_2.5_ Carbon monoxide	The study reported respiratory symptoms in pregnant women. Among the respiratory symptoms were increased risk of pneumonia and severe pneumonia.
Boamah- Kaali et al. (2021) [31]	Prenatal and Postnatal Household Air Pollution Exposure and Infant Growth Trajectories: Evidence from a Rural Ghanaian Pregnancy Cohort	Quantitative method	pregnant women (N = 1306)	Ghana	Carbon monoxide PM_2.5_	Associated with poor infant growth.
Daouda et al. (2024) [32]	Prenatal Household Air Pollution Exposure and Childhood Blood Pressure in Rural Ghana	Quantitative method	pregnant women (N = 1414)	Ghana	Carbon monoxide PM_2.5_	The study highlighted that the exposure to HAP during perinatal and first year of life increases the blood pressure among children.

**Table 3:** Countries and characteristics of the included studies.

Items	Responses	Frequency (N) (%)
Country	Ethiopia	2 (10.5%)
	Ghana	7 (36.8%)
	Nigeria	4 (21.1%)
	South Africa	4 (21.1%)
	Tanzania	2 (10.5%)
Total		19 (100%)
Research Methodology	Qualitative	2 (10.5%)
	Quantitative	16 (84.2%)
	Mixed method	1 (5.3%)
Total		19 (100%)

## Data Availability

No new data was created or analyzed in this review. Data sharing is not applicable to this research and required data is cited in this review.
